# Survival with AGS-003, an autologous dendritic cell–based immunotherapy, in combination with sunitinib in unfavorable risk patients with advanced renal cell carcinoma (RCC): Phase 2 study results

**DOI:** 10.1186/s40425-015-0055-3

**Published:** 2015-04-21

**Authors:** Asim Amin, Arkadiusz Z Dudek, Theodore F Logan, Raymond S Lance, Jeffrey M Holzbeierlein, Jennifer J Knox, Viraj A Master, Sumanta K Pal, Wilson H Miller, Lawrence I Karsh, Irina Y Tcherepanova, Mark A DeBenedette, W Lee Williams, Douglas C Plessinger, Charles A Nicolette, Robert A Figlin

**Affiliations:** Levine Cancer Institute, Charlotte, NC USA; University of Illinois Cancer Center, Chicago, IL USA; Indiana University Simon Cancer Center, Indianapolis, IN USA; Urology of Virginia, Norfolk, VA USA; University of Kansas Medical Center, Kansas City, KS USA; Princess Margaret Hospital, Toronto, ON Canada; Emory University, Atlanta, GA USA; City of Hope Comprehensive Cancer Center, Duarte, CA USA; Lady Davis Institute and Segal Cancer Center-Jewish General Hospital, McGill University, Montreal, QC Canada; The Urology Center of Colorado, Denver, CO USA; Argos Therapeutics, Inc., Durham, NC USA; Cedars-Sinai Medical Center, Los Angeles, CA USA

**Keywords:** Immunotherapy, Dendritic cell, RCC, AGS-003, Sunitinib

## Abstract

**Background:**

AGS-003 is an autologous immunotherapy prepared from fully matured and optimized monocyte-derived dendritic cells, which are co-electroporated with amplified tumor RNA plus synthetic CD40L RNA. AGS-003 was evaluated in combination with sunitinib in an open label phase 2 study in intermediate and poor risk, treatment naïve patients with metastatic clear cell renal cell carcinoma (mRCC).

**Methods:**

Twenty-one intermediate and poor risk patients were treated continuously with sunitinib (4 weeks on, 2 weeks off per 6 week cycle). After completion of the first cycle of sunitinib, patients were treated with AGS-003 every 3 weeks for 5 doses, then every 12 weeks until progression or end of study. The primary endpoint was to determine the complete response rate. Secondary endpoints included clinical benefit, safety, progression free survival (PFS) and overall survival (OS). Immunologic response was also monitored.

**Results:**

Thirteen patients (62%) experienced clinical benefit (9 partial responses, 4 with stable disease); however there were no complete responses in this group of intermediate and poor risk mRCC patients and enrollment was terminated early. Median PFS from registration was 11.2 months (95% CI 6.0, 19.4) and the median OS from registration was 30.2 months (95% CI 9.4, 57.1) for all patients. Seven (33%) patients survived for at least 4.5 years, while five (24%) survived for more than 5 years, including 2 patients who remain progression-free with durable responses for more than 5 years at the time of this report. AGS-003 was well tolerated with only mild injection-site reactions. The most common adverse events were related to expected toxicity from sunitinib therapy. In patients who had sequential samples available for immune monitoring, the magnitude of the increase in the absolute number of CD8^+^ CD28^+^ CD45RA^−^ effector/memory T cells (CTLs) after 5 doses of AGS-003 relative to baseline, correlated with overall survival.

**Conclusions:**

AGS-003 in combination with sunitinib was well tolerated and yielded supportive immunologic responses coupled with extension of median and long-term survival in an unselected, intermediate and poor risk prognosis mRCC population.

**Clinical Trial Registry:**

#NCT00678119

## Background

Renal cell carcinoma (RCC) is an immunologically responsive tumor and until recently, the mainstay for systemic therapy for advanced metastatic RCC was cytokine-based immunotherapy with interleukin 2 (IL-2) and interferon alpha (IFN-α). During the past decade, identification of critical cellular growth factor pathways in RCC has enabled development of drugs targeting the vascular endothelial growth (VEGF) pathway and the mammalian target of rapamycin (mTOR) complex. Approved VEGF tyrosine kinase inhibitors (TKIs) and mTOR inhibitors have shown clinically relevant benefit in phase 3 trials [1]. Although durable responses are rarely seen with these targeted agents [1-3], they can be used for a broader spectrum of patients than cytokines and the clinical benefit as evidenced by improvements in both progression-free and overall survival, primarily in patients with favorable and intermediate risk profiles, has positively impacted the natural history of metastatic RCC (mRCC) [4].

In contrast to the VEGF and mTOR inhibitors, durable and complete responses have been observed with high dose (HD) IL-2, indicating the importance of immune modulation in this disease process [5]. While 25% may show response, only 5-8% of patients experience durable complete responses. HD IL-2 is, however, associated with significant toxicity, which has limited this modality to be delivered at specialized centers in a highly selected and limited population of mRCC patients with a high level of cardiopulmonary fitness [6]. Immune modulation, if it can be delivered with low toxicity and be applicable to a broader mRCC population, would be a desirable therapeutic approach to pursue in combination with standard targeted therapy.

Dendritic cells (DCs) are a powerful tool for stimulating cell-mediated immunity by efficient presentation of antigen to both CD4^+^ and CD8^+^ T cells [7,8]. AGS-003 is an autologous immunotherapy approach which provides the critical signals required to generate a patient and tumor specific adaptive immune response. AGS-003 is prepared ex vivo from matured monocyte-derived DCs co-electroporated with the patient's amplified tumor RNA and synthetic CD40L RNA [9-12]. When administered by intradermal injection, these optimized, RNA-loaded mature DCs are capable of presenting the relevant patient-specific tumor antigens, via MHC-Class I presentation, to T cells in the draining lymph node basin. Additionally, CD40 ligation optimizes CD8^+^ T cell induction through production of IL-12 [11,12]. AGS-003 has previously been evaluated as a monotherapy [13] in newly diagnosed, intermediate and poor risk, synchronous mRCC patients and was tolerated well with no grade 3 or 4 adverse events. In a predominantly poor risk population, median PFS was 5.5 months and median overall survival (OS) was 15.7 months, with 23% of patients surviving for 3.5 to 8 plus years, despite limited use of any subsequent systemic therapy, including targeted therapy (T. Logan, A. Amin, V. Cohen, et al. A Phase 1/2 Study of AGS-003, a personalized immunotherapeutic evaluated in newly diagnosed metastatic renal cell carcinoma subjects; In preparation). The use of autologous tumor RNA for broad tumor antigen presentation by autologous, mature DCs holds promise as a fully personalized, patient-specific immunotherapeutic product as it minimizes the risk of mutant clonal escape with presentation of multiple target antigens from the autologous tumor sample [14-17].

Sunitinib is the standard of care for first-line treatment of mRCC. It is particularly attractive for combination therapy with a novel immunotherapeutic approach. Sunitinib has been shown to elicit a positive modulatory effect on the immune system through suppression of myeloid-derived suppressor cells (MDSCs) and T regulatory (Treg) cells [18,19]. Tumors are infiltrated with Treg cells and MDSCs that actively inhibit T-cell responses and contribute to altered immune surveillance in RCC [20,21]. While targeted therapies such as sunitinib have yielded improved efficacy over the past decade, durable remissions and long-term survival are rare, particularly in newly diagnosed, intermediate and poor risk metastatic RCC (mRCC) patients. A recent analysis by the International mRCC Database Consortium (IMDC) indicates newly diagnosed, intermediate and poor risk mRCC patients who present with the time from diagnosis to initiation of treatment less than 1 year risk factor (DxTx < 1y), have an expected median progression-free survival (PFS) of 5.6 months and median overall survival (OS) of 14.7 months, despite treatment with standard targeted therapy [22]. When treated with sunitinib alone, the expected percentage of intermediate and poor risk mRCC patients surviving greater than 30 months is 13% [23].

The Phase 2 study was designed to assess the efficacy and safety of AGS-003 in combination with sunitinib in patients with newly diagnosed, intermediate and poor risk, synchronous mRCC after nephrectomy.

## Results

### Patients

**Figure 1:**
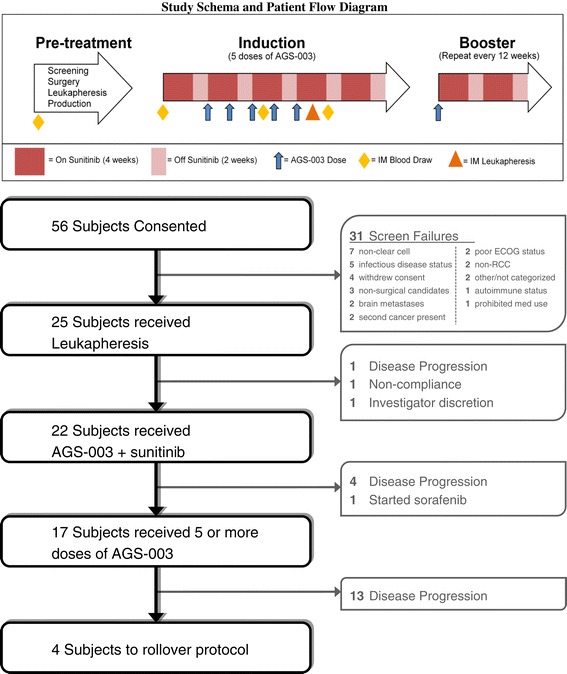
**Study Schema and Patient Flow Diagram.**

Of 25 enrolled patients who underwent leukapheresis, three patients were withdrawn prior to study treatment, either due to rapid disease progression (n = 1), uncontrolled hypertension (n = 1) or withdrawal of consent (n = 1) (Figure 1). Twenty two patients entered the induction phase and received sunitinib and at least one dose of AGS-003, including one patient who had enrolled in a prior AGS-003 study. This rollover patient was excluded from efficacy evaluations and included for safety evaluation only.

Demographics and tumor characteristics for the efficacy population (n = 21) are shown in Table 1. Median patient age was 56 years (range 22-68), all treated patients had clear cell mRCC, and the majority were Fuhrman grade 3 or 4 (76%, 16/21) and were T3 or T4 (72%, 15/21). All study patients presented with synchronous, measurable metastatic disease and had a time from diagnosis to treatment of less than 1 year (mean = 10.8 ± 6.41 weeks). Patients were classified as intermediate (71%) or poor (29%) risk according to Memorial Sloan Kettering Cancer Center (MSKCC) [24] and intermediate (52%) or poor (48%) risk according to Heng criteria [25].

**Table 1:** **Demographics and Patient RCC Characteristics**

**Characteristics**	**Patients ** **N = 21**
Age, median years (range)	56.0 (22-68)
**Sex, n (%)**	
Male	16 (76)
Female	5 (24)
**Race, n (%)**	
Caucasian	19 (91)
Black	1 (5)
Asian	1 (5)
**Time from diagnosis to 1** ^st^ **sunitinib dose, mean weeks (SD)**	10.8 (6.41)
**ECOG performance status, n (%)**	
0	15 (71)
1	6 (29)
**MSKCC risk [** 23 **], n (%)**	
Favorable (0 risk factors)	0
Intermediate (1-2 risk factors)	15 (71)
Poor (≥3 risk factors)	6 (29)
**Heng risk [** 24 **], n (%)**	
Favorable (0 risk factors)	0
Intermediate (1-2 risk factors)	11 (52)
Poor (≥3 risk factors)	10 (48)
**Fuhrman nuclear grade, n (%)**	
Grade 2	5 (24)
Grade 3	9 (43)
Grade 4	7 (33)
**Tumor Size, n (%)**	
TX	2 (10)
T1	3 (14)
T2	1 (5)
T3	13 (62)
T4	2 (10)
**Regional Lymph Nodes Staging, n (%)**	
NX	10 (48)
N0	6 (29)
N1	1 (5)
N2	4 (19)
**Distant Metastasis Staging, n (%)**	
M1	21 (100)

ECOG = Eastern Cooperative Oncology Group MSKCC = Memorial Sloan Kettering Cancer Center; n = number of patients; SD = standard deviation.

### AGS-003 Production, Characteristics, and Patient Exposure

The median time from leukapheresis to production and administration of the first AGS-003 dose was 7 weeks. The median time from nephrectomy/metastasectomy to first dose was 11 weeks. The median number of AGS-003 doses administered was 6 (2-15). Nine patients (43%) received eight or more AGS-003 doses. Eight patients (38%) had sunitinib dose delays or dose reductions during the trial, including one patient who discontinued sunitinib while on study. Overall, 10 (48%) patients received subsequent therapy after progression, including TKIs (n = 6), chemotherapy (n = 4), mTOR inhibitor (n = 2), cytokine therapy (n = 1) and investigational immunotherapy (n = 1).

### Clinical Responses

#### Tumor Responses

The best overall tumor response per RECIST, after at least one 6-week cycle of sunitinib, are shown in Table 2. No complete responses (CRs) were observed. Overall, 43% (9/21) of patients experienced a partial response (PR) as their best response (2 PRs were observed following the initial 6-week cycle of sunitinib). In addition, 4 PRs were observed during induction with AGS-003 plus sunitinib, while 3 additional PRs developed during the prolonged booster phase, more than 1 year after initiating combined treatment. Two of these patients continue on study treatment for more than 5 years at the time of this report. Additionally, 4 patients experienced stable disease. The overall clinical benefit rate for the combination (CR + PR + stable disease [SD]) was 62% (13/21).

**Table 2:** **Tumor response data**

Best overall response*	**PR**	**SD**	**PR + SD**	**PD**
	**N (%)**			
**All patients (n = 21)**	5 (24)	8 (38)	13 (62)	8 (38)
**MSKCC intermediate risk (n = 15)**	4 (19)	7 (33)	11 (52)	4 (19)
**MSKCC poor risk (n = 6)**	1 (5)	1 (5)	2 (10)	4 (19)
**Heng intermediate risk (n = 11)**	4 (19)	5 (24)	9 (43)	2 (10)
**Heng poor risk (n = 10)**	1 (5)	3 (14)	4 (19)	6 (29)

*Restaging scans occurred after five doses of AGS-003 and cycles 2-4 of sunitinib. NOTE: Responses were determined relative to a baseline scan conducted after nephrectomy and completion of one (1) 6-week cycle of sunitinib.

PD = progressive disease; PR = partial response; SD = stable disease.

#### Progression-Free Survival

Median PFS (95% CI) was 11.2 months (6.0, 19.4). Based upon the Heng risk criteria, the median PFS was 5.8 (4.3, 11.2) months for poor risk and 19.4 (7.2, 38.1) months for intermediate risk patients. The Kaplan-Meier PFS estimates for all patients and by Heng risk group are shown in Table 3.

**Table 3:** Kaplan-Meier estimates of progression free survival for all patients and for patients in Heng intermediate and poor risk factor groups*

**Patient group**	**Number of patients at risk**													**Median time to progression or death Months (95% CI)**
**Month**	0	3	6	9	12	15	18	21	24	27	30	33	36	
**All patients (n = 21)**	21	21	15	12	10	8	7	6	4	4	3	1	1	11.2 (6, 19.4)
**Heng intermediate risk group (n = 11)**	11	11	11	9	8	6	6	5	4	4	3	1	1	19.4 (7.2, 38.1)
**Heng poor risk group (n = 10)**	10	10	4	3	2	2	1	1	0	0	0	0	0	5.8 (4.3, 11.2)

*Similar results were obtained for MSKCC intermediate (14.9 months) and poor (5.7 months) risk factor groups.

CI = confidence interval.

#### Overall Survival

**Figure 2:**
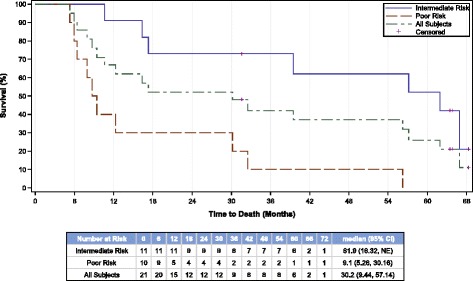
**Overall survival.** Kaplan-Meier estimates of overall survival (months) for all patients and by patients in Heng intermediate and poor risk factor groups. Similar results were obtained for MSKCC intermediate (39.5 months) and poor (7.9 months) risk factor group.

Median OS (95% CI) for all patients was 30.2 (9.4, 57.1) months. Based on the Heng risk criteria, the estimated median OS was 61.9 months (16.3, NE) for intermediate risk and 9.1 (5.3, 30.2) months for the poor risk patients. The Kaplan-Meier OS estimates for all patients and by Heng risk group are shown in Figure 2.

Overall, 52% (11/21) of patients experienced long-term survival > 30 months. At the time of this report, 7 (33%) patients are alive more than 4.5 years from study registration, while 5 (24%) are alive for more than 5 years, including two intermediate risk patients who remain progression-free and continue to receive booster AGS-003 dosing and reduced-dose sunitinib on a separate protocol.

### Immune Responses

**Figure 3:**
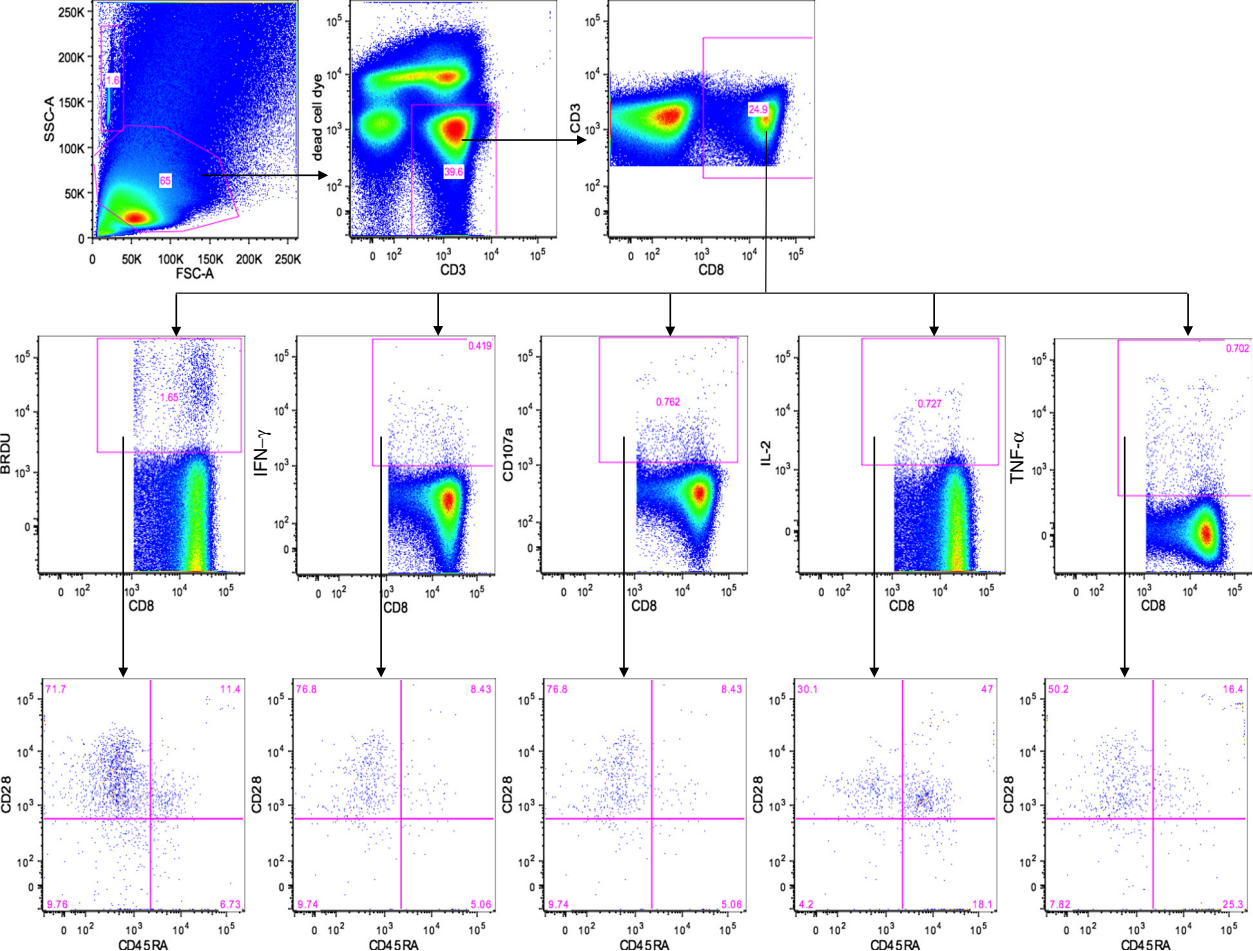
**Representative flow cytometry plots detailing the gating strategy to identify CD28+/CD45RA- CD8 + T cells expressing effector molecules.** The viable CD3^+^ CD8^+^ T cells in the lymphoycte gate were identified and futher subgated to analyze CD28 and CD45RA expression on the Brdu^+^, IFN-g^+^ CD107a^+^ IL-2^+^ and TNFa^+^ cells within the CD8^+^ T cell gate. The number of cellular events in the CD28+/CD45RA- upper left quadrant were used to determined the absolute number of cells/ml. Identical gates were used for all samples analyzed.

**Figure 4:**
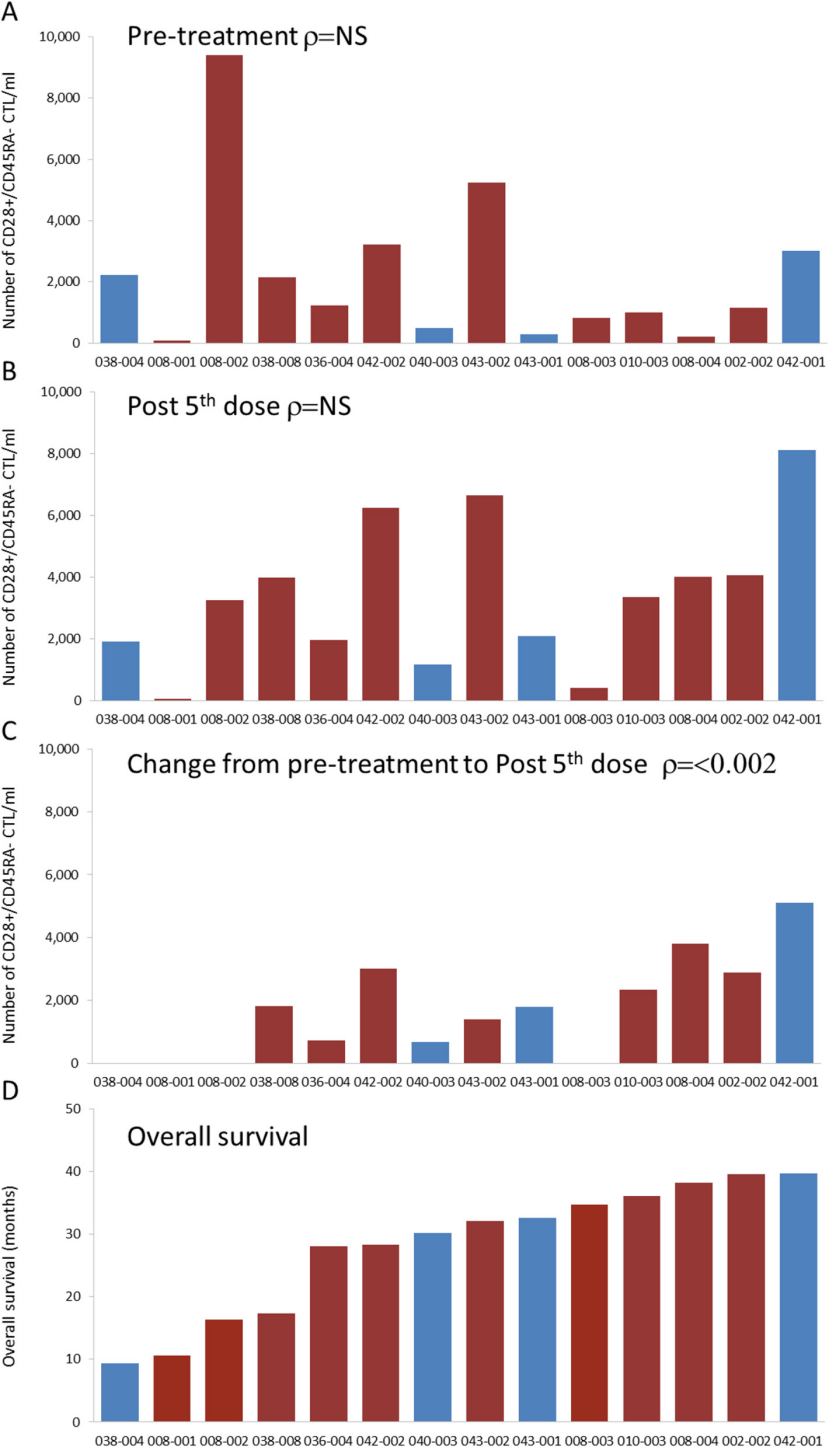
**Measurement of AGS-003 induced multifunctional CD8** ^+^ **CD28** ^+^ **CD45RA** ^−^ **CTLs and correlation with overall survival.** Absolute number of CD28+/CD45RA- CTLs in response to DCs electroporated with autologous amplified RCC tumor RNA at baseline **(A)** and after 5 doses of AGS-003 **(B)**. Increase in the number of CD28+/CD45RA- CTL from baseline to post 5^th^ dose **(C)**. Patients are listed by in increasing order of overall survival **(D)**. Blue bars () identify intermediate risk patients and red bars () identify poor risk patients. The correlation between the absolute number of CD28+/CD45RA- CTLs and overall survival was statistically significant by nonparametric bivariate analysis, Spearman’s ρ = 0.8; p < 0.002.

Fourteen of 21 patients received 5 doses of AGS-003, underwent a subsequent leukapheresis, and had evaluable samples for immunologic analyses. Preclinical studies demonstrated that AGS-003 administration results in the generation of CD8^+^CD28^+^CD45RA^−^ effector memory CTLs. Previous published work revealed that in vitro priming with post matured CD40L RNA electroporated DCs encoding specific target antigen expanded multi-functional antigen specific CTLs exhibiting an effector memory phenotype defined by the expression of CD28 and negative for CD45RA expression. Therefore the primary immune monitoring end point analysis focused on identifying increases in the numbers of functional CTLs expressing CD28 in the absence of CD45RA after administration of AGS-003 [11,12]. A CTL was considered functional if it expressed any of the functional markers defined by cytokine secretion (IFN-g, TNF-a, and IL-2), lytic function (CD107a), or proliferated (Brdu). Figure 3 outlines the gating strategy to detect functional CD28+/CD45RA- CTLs. Functional CD28+/CD45RA- CTLs were quantitated prior to systemic therapy (post-nephrectomy prior to sunitinib) and after 5 doses of AGS-003 in combination with sunitinib. The baseline level for each patient was determined by averaging the absolute number of functional CD28+/CD45RA- CTLs/ml after in vitro stimulation for the two baseline samples, one pre- and one post-nephrectomy, both prior to AGS-003 administration. The absolute numbers of functional CD28+/CD45RA- CTLs were determined after the 5^th^ dose of AGS-003. Neither the baseline response prior to AGS-003 administration (Figure 4A) nor the response after the 5^th^ dose of AGS-003 (Figure 4B) correlated with overall survival (Figure 4D). However after the 5^th^ dose, 10 of 14 (71%) patients analyzed displayed an increase in the number of functional CD28+/CD45RA- CTLs over baseline (Figure 4C). Furthermore, this change in the magnitude of the response showed a statistically significant correlation with the duration of survival (nonparametric bivariate analysis, Spearman’s ρ = 0.8; p < 0.002). Therefore the detection of newly generated CTLs are most likely to be tumor antigen-specific following stimulation with autologous DCs electroporated with autologous amplified tumor RNA. Notably, one patient exhibited prolonged survival in the absence of a detectable increase in the absolute number of CTLs. Interestingly, this patient had significant numbers of CTLs at baseline, prior to AGS-003 initiation.

### Safety

Treatment emergent adverse events occurring in five or more patients are listed in Table 4. Table 4 also shows the relationship of adverse events to study treatments (AGS-003, sunitinib or combination). All patients reported at least one event. There were no grade 4 toxicities reported, and no patient withdrew from the study due to an adverse event. Nine patients had grade 3 events attributed to sunitinib treatment; none of these were attributed to AGS-003 treatment. There were seven treatment emergent serious adverse events reported (one case each of bradycardia, constipation, diarrhea, pneumonia, dehydration, hypocalcemia and urinary retention), that resolved without sequelae with standard management. No grade 3 or serious adverse event was considered related to AGS-003. There were no treatment related deaths.

**Table 4:** Treatment emergent adverse events occurring in 5 or more patients and relationships* to study drug treatment

**Adverse event**	**Treatment-Emergent adverse events ** **N = 22 ** **Number of patients (%)**			
	**All**	AGS-003 related**	**Sunitinib related**	**Combination related**
**Any**	22 (100)	17 (77)	22 (100)	12 (55)
**Diarrhea**	13 (59)	3 (14)	11 (50)	3 (14)
**Fatigue**	13 (59)	3 (14)	12 (55)	2 (9)
**Nausea**	12 (55)	2 (9)	11 (50)	2 (9)
**Rash**	10 (46)	4 (18)	9 (41)	5 (23)
**Decreased weight**	9 (41)	1 (5)	5 (23)	1 (5)
**Headache**	8 (36)	2 (9)	4 (18)	2 (9)
**Injection-site erythema**	8 (36)	7 (32)	0	0
**Peripheral edema**	8 (36)	0	4 (18)	0
**Dyspnea**	7 (32)	0	1 (5)	0
**Vomiting**	7 (32)	0	7 (32)	0
**Anorexia**	6 (27)	0	5 (23)	1 (5)
**Hypertension**	6 (27)	1 (5)	6 (27)	1 (5)
**Hypothyroidism**	6 (27)	0	5 (23)	1 (5)
**Hand-foot syndrome**	6 (27)	0	6 (27)	0
**Constipation**	5 (23)	0	2 (9)	0
**Dehydration**	5 (23)	0	2 (9)	0
**Dysgeusia**	5 (23)	1 (5)	5 (23)	1 (5)
**Injection-site induration**	5 (23)	5 (23)	0	1 (5)

*Events described as possible, probably or definitely related to study drug.

**All AGS-003 related events were Grade 1 or 2 in severity.

The most frequent AGS-003-related events were mild (grade 1) injection-site reactions, including erythema (7/22 patients), induration (5/22), swelling (4/22) pain (3/22) and pruritus (3/22). No adverse events unique to the combination of AGS-003 plus sunitinib were reported.

Adverse events reported within 24 hours of a leukapheresis procedure occurred in eleven patients, including vomiting in two patients (one of whom also had nausea and decreased weight), four patients with pain, three patients with fatigue, one patient with constipation and nausea, and one patient with muscle spasms and oral paresthesia. All were mild to moderate in severity and did not preclude further participation nor require hospitalization.

No evidence of emergent autoimmunity was noted per laboratory assessments for auto-immune markers. There were no clinically relevant outcomes for hematology, biochemistry, and urinalysis laboratory assessments, physical examinations, vital signs, or ECGs.

## Discussion

During the past decade, VEGF-targeted therapies have become standard treatment for advanced RCC. While targeted therapies have yielded improved efficacy, durable remissions, CRs (<1%) and long-term survival (>30 months) are rare, particularly in intermediate and poor risk metastatic RCC (mRCC) patients [25,26]. A recent analysis by the International mRCC Database Consortium (IMDC) revealed that newly diagnosed, intermediate and poor risk mRCC patients who present with the time from diagnosis to initiation of treatment less than 1 year risk factor (DxTx < 1y), have an expected median PFS of 5.6 months and median OS of 14.7 months, despite treatment with standard, sequential targeted therapy [22]. Attempts to improve outcome using combinations of targeted agents including TKIs, mTORs, plus interferon have been limited due to severe and/or overlapping toxicity [27-30]. While immune modulation with IL-2 has shown durable responses, it is limited to a select population of patients. Therefore, there is a need to identify novel immunomodulatory therapies for mRCC.

The rationale to combine sunitinib with AGS-003, a fully personalized immunotherapy, was based upon the proven antitumor activity of sunitinib and its potential to attenuate the immune suppression in the tumor micro-environment observed in advanced RCC [18,19]. In this study, AGS-003 was evaluated in combination with sunitinib for the treatment of newly diagnosed mRCC patients with intermediate or poor risk as defined by either MSKCC or Heng criteria. All patients presented with the DxTx < 1 yr risk factor and the majority presented with at least 2, 3 or 4 risk factors. When this study was initially designed, the primary objective was to determine whether the addition of AGS-003 to sunitinib could achieve a 20% complete remission rate. As TKI data matured, it became evident that sunitinib was rarely if ever associated with CRs in advanced RCC, even in studies where approximately one-third of subjects entered with favorable risk disease [26,31,32]. Based on emerging data and insights during study conduct, it became evident that the primary endpoint was inappropriate for this population. Therefore, further enrollment was stopped. An independent safety monitoring board established at study start continued to monitor safety for the duration of the study. Patients already enrolled continued treatment and follow-up per protocol to assess secondary endpoints such as PFS, OS and safety. These endpoints were deemed to be much more instructive for future AGS-003 development efforts in similar, newly diagnosed intermediate and poor risk mRCC patients.

In addition to limited expectations for durable complete remissions with targeted therapies such as sunitinib, these treatments also appear to exert both cytotoxic and cytostatic effects which complicate response assessment, particularly when combined with immunotherapy. RECIST-based criteria used traditionally for evaluating response to cytotoxic agents may therefore not be adequate or helpful to evaluate clinically relevant outcomes with targeted therapies alone, since they may induce necrosis with minimal change in size with conventional imaging [33]. Yet another layer of complexity regarding response evaluation is added by the immunotherapy component used in combination with sunitinib in this study. Observations with immunotherapy in patients with metastatic melanoma treated with agents such as ipilimumab, led to the development of immune response criteria that showed disease stability translates into clinical benefit. Delayed responses were noted even after initial increases in tumor burden [34]. Therefore, disease stabilization and overall survival may be more appropriate measures of clinical benefit with new immunotherapeutic modalities such as AGS-003. This is further supported by the proportion of melanoma patients treated with ipilimumab demonstrating durable, long term overall survival at a follow-up of 5 to 6 years [35].

In this study, the median PFS for all subjects was 11.2 months and the median OS was 30.2 months, which represents a near doubling of expected survival in this group of patients. The median OS was estimated to be more than 5 years at 61.9 months for the 11 patients classified as Heng intermediate risk (1-2 risk factors [22]) which are encouraging and suggest that combined targeted therapy plus immunotherapy may have a significant impact on survival outcomes in intermediate risk mRCC patients.

Fifty-two percent of the evaluable patients receiving AGS-003 plus sunitinib demonstrated long term (>30 month) survival. This compares favorably with historical analysis indicating approximately 13% of intermediate and poor risk mRCC patients treated with sunitinib alone survive for > 30 months, which has been defined as long-term in the advanced RCC setting [25]. In addition, a third of the patients on this study were alive and in follow-up or continuing treatment with AGS-003 plus sunitinib for more than 4.5 years. As of June 2014, 24% (5/21) had survived for more than 5 years after study initiation, including 2 patients in prolonged partial remissions who continue AGS-003 booster dosing plus reduced-dose sunitinib.

The intended mechanism of action of AGS-003 is to induce CD8^+^ CD28^+^ CD45RA^−^ CTLs against patient-specific tumor antigens. Under normal conditions, activated helper CD4^+^ T cells up-regulate CD40L and physically interact with DCs resulting in effective CD8^+^ cytotoxic T cell responses [36]. However, mRCC patients are immunosuppressed due to local and systemic influences of the tumor cells and display both DC and CD4^+^ helper T cell dysfunction [19,37-39]. AGS-003 addresses the immune dysfunction of the host by co-electroporation of RNA encoding CD40L into the *ex vivo*-prepared DCs to simulate the presence of CD4^+^ T cell help via the intracellular ligation of endogenous CD40 within the DCs. This also results in the secretion of IL-12 from the DCs, a requirement for the generation of T cell responses [11]. Additional functionality was engineered into AGS-003 via the novel method by which the cells are matured. Rather than employing the traditional ‘cytokine cocktail’ method [40], sequential cytokine exposure was employed which results in DCs that primarily induce CTL generation [10]. This functionality is important because memory T cell responses have been associated with good clinical outcome in patients with solid tumors [41-43]. Collectively, these properties allow AGS-003 to generate anti-tumor memory T cell responses within the immunosuppressed patient.

The increase in the absolute number of CTLs between baseline and fifth dose of AGS-003 was a statistically significant correlate to survival, even with this small number of patients (Figure 4C and D). The number of tumor-reactive CTLs prior to (baseline) and after the 5^th^ dose of AGS-003 did not correlate with survival (Figure 4A and B). This suggests that the pre-existing T cells were not contributing to clinical outcome and only the newly generated, effector memory CTLs induced by AGS-003 administration were functionally significant. A positive increase in the magnitude of the induced CTL response is indicative of the induction of an antigen specific CTL response following AGS-003 administration. It is to be noted that these data were obtained prospectively since the clinical outcomes were not known at the time the immunologic assessment was carried out. The prognostic value of absolute changes in CTL numbers in response to AGS-003 as an early biomarker for overall survival is being prospectively assessed in the ongoing pivotal ADAPT Phase 3 clinical trial (Clinical Trial Registry #NCT01582672).

In clinical practice, the most common side effects of sunitinib treatment include fatigue/asthenia, anorexia/loss of appetite, hypothyroidism, hand-foot syndrome, stomatitis/taste changes, diarrhea/abdominal pain, myelosuppression, and hypertension [44]. The most common adverse events in the present study were consistent with those reported with sunitinib alone in advanced RCC. Adverse events attributed to AGS-003 were primarily mild injection-site reactions, which occurred in about half of the patients. No adverse events unique to the combination of AGS-003 plus sunitinib were reported. No grade 4 events occurred during the study and no grade 3 events considered related to AGS-003 therapy. These safety results are noteworthy, since many newly approved targeted therapies are associated with serious side effects (gastrointestinal, skin, and vascular events) that affect morbidity and mortality and limit the ability to combine these newer treatments [45].

## Conclusion

When compared to outcomes and benchmarks established with targeted therapy, the addition of AGS-003 to sunitinib in an unselected, intermediate and poor risk mRCC patient population was associated with a doubling of expected survival, encouraging long-term and 5-year overall survival, and an excellent safety profile. In addition, the target effect of AGS-003, an expansion of effector memory CTLs, was observed after 5 doses and correlated to prolonged survival. These encouraging findings support the ongoing phase 3, randomized ADAPT study, which has been designed to compare the addition of AGS-003 with standard surgery and targeted drug therapy to standard surgery and targeted drug therapy alone in newly diagnosed, intermediate and poor risk mRCC patients.

## Methods

The trial was a single-arm, open-label phase 2 study conducted from January 2008 to February 2012 at 10 centers in North America (Clinical Trial Registry #NCT00678119). Treatment was administered according to International Conference on Harmonization Good Clinical Practice guidelines and applicable local regulatory requirements and laws, and the clinical protocol was approved by institutional review boards or independent ethics committees at each study center. All patients provided written informed consent.

### Patients

Men and women at least 18 years of age with newly or recently diagnosed synchronous, metastatic RCC (mRCC) and predominantly clear cell tumor were enrolled if they had no prior nephrectomy or had a recent nephrectomy, but had at least one accessible metastatic lesion for metastasectomy. Inclusion criteria required measurable metastatic disease per Response Evaluation Criteria in Solid Tumors (RECIST) [46] and Eastern Cooperative Oncology Group (ECOG) performance status 0 or 1. All patients included had a time from diagnosis to treatment of less than 1 year (DxTx < 1 yr).

Patients were required to be candidates for sunitinib therapy and were required to have adequate end organ function. Patients with brain metastases, uncontrolled hypertension, Type 1 diabetes, active autoimmune disease, or previous systemic therapy for advanced RCC were excluded.

### AGS-003 Production

AGS-003 was manufactured at a centralized GMP compliant facility (Argos Therapeutics, Durham, NC). Following screening and consent, autologous tumor total RNA was isolated from nephrectomy or metastasectomy tissue samples and messenger RNA was amplified using RT/PCR and *in vitro* transcription technologies as previously described [47]. CD40L RNA was manufactured using *in vitro* transcription and a post-transcriptional capping method [48]. Patients had leukapheresis at the clinical site’s donor center using a COBE Spectra® Leukapheresis System (Gambro BCT, Lakewood, CO). Monocytes were cultured in AIM-V media with 800 U/mL granulocyte macrophage-colony stimulating factor (Berlex) and 1000 U/mL IL-4 (R&D Systems) to generate immature DCs that were then matured using 20 ng/mL tumor necrosis factor alpha (TNF α) (R&D Systems)/1000 U/mL IFN-γ (InterMune)/1 μg/mL prostaglandin E2 (Sigma). Mature DCs were electroporated with the amplified tumor RNA and CD40L RNA using a post-maturation electroporation protocol [10].

The final AGS-003 product was formulated as 1.4 × 10^7^ DC/0.7 mL in 80% autologous plasma, 10% dextrose (50% w/v) (Hospira), and 10% DMSO (Sigma) and cryopreserved in liquid nitrogen vapor phase. Thawed samples of final product were assessed for sterility, mycoplasma, endotoxin, and viability prior to release for clinical use.

### Treatment

The treatment schedule is illustrated in Figure 1. Prior to initiation of AGS-003 therapy, patients initiated sunitinib therapy on a standard, repeating 6-week cycle of 50 mg daily for 4 weeks followed by 2 week rest. Dose modifications (reductions, delays, and/or discontinuation due to toxicity) for sunitinib were permitted per standard labeling throughout study directed treatment. AGS-003 was administered prior to the initiation of the second 6-week cycle of sunitinib (week 6). Each dose of AGS-003 consisted of 1.2 × 10^7^ DCs delivered as three intradermal injections of 0.2 mL (0.6 mL total) to the axillary lymph node basin. AGS-003 treatment continued every 3 weeks for a total of five doses (induction phase) in combination with sunitinib. Following induction, AGS-003 was administered every 12 weeks along with standard sunitinib (booster phase). Treatment was continued until disease progression, intolerable toxicity to standard of care, or end of study. Patients who continued to benefit from AGS-003 treatment at the time of study closure were rolled over to companion AGS-003 trials.

### Tumor Response Assessments

Tumor measurements were assessed pre-nephrectomy/metastasectomy, after one cycle of sunitinib prior to the initiation of AGS-003, and after the fifth dose of AGS-003. During booster treatment, imaging occurred every 12 weeks.

### Immune Response Assessments

Preparation of blood draw samples for immune monitoring by multi-color flow cytometry. Frozen PBMCs processed by ficoll density gradient separation from whole blood draws were collected for immune monitoring prior to surgery, prior to initiation of sunitinib, and at two points after initiation of AGS-003, following the third and fifth AGS-003 dose (Figure 1). PBMCs were thawed and rested overnight in X-Vivo 15 supplemented with 10% Human AB serum. After overnight rest PBMCs were labeled with BRDU (bromodeoxyuridine) to track T-Cell proliferation. A leukapheresis for immune monitoring was collected after the fifth dose and autologous DC targets for in vitro stimulation were prepared from DCs co-electroporated with CD40L RNA and autologous RCC tumor RNA for each evaluable subject. Autologous cultures containing DCs and PBMCs were setup and incubated at 37°C for 6 days. On day 6, cultures were re-stimulated with autologous DCs prepared as stated above and anti-CD107a antibody was added to each tube and incubated at 37°C for 5 hours in the presence of Brefeldin A (BD Biosciences). After incubation, cells were stained for viability using annexinV and a viability dye (Invitrogen), which permits selection of viable cells, followed by surface staining with specific antibodies for detection of CD28, CD45RA, CD3, and CD8 expression. After surface staining, cells were fixed with 4% BD Cytofix and stored overnight in BSA staining buffer at 4°C. The following day, the cells were permeabilized and DNase treated for 1 hour at 37°C using reagents included in the BRDU staining kit (BD biosciences). Intracellular staining for IFN-γ, TNF-α, IL-2, and BRDU were performed. After staining, cells were washed and diluted in 500 μl BSA buffer and transferred to a BD TruCount Tube for acquisition on a BD LSRII cytometer. 400,000-600,000 events were collected per sample. Number of cells/ml was calculated using the following formula; (number of cellular events collected/number of beads collected) x (bead concentration)/collected volume) × 1000.

### Gating strategy to define the absolute numbers of functional CD28+/CD45RA- CTLs

The absolute number of cells expressing IFN-g, IL-2, TNF-a, CD107a or BRDU were first identified in the CD8+ viable cell gate. Within each gate identifying the number of IFN-g, IL-2, TNF-a, CD107a or BRDU positive cells, the number of cells positive for CD28 and negative for CD45RA were subsequently determined (Figure 3). To determine the total number of functional CD28+/CD45RA- CTLs, the numbers of CD28+/CD45RA- CTLs having any function were added together to determine the absolute number of functional CD28+/CD45RA- CTLs. This was done for each subject at each indicated time point, pre and post 5^th^ dose AGS-003 administration. To define the overall magnitude for the response to autologous AGS-003 product the value determined pre AGS-003 administration was subtracted from the value determined after the 5^th^ AGS-003 dose administration to calculate the absolute change in CTLs between these two time points.

### Endpoints

The efficacy and safety population consisted of all study subjects who received at least one AGS-003 dose. One patient enrolled on a previous AGS-003 monotherapy study was included in the safety, but not efficacy population because of prior AGS-003 exposure.

The primary endpoint was the CR rate as defined by RECIST v1.0 [46]. Secondary endpoints included PFS, OS, immune response and clinical benefit (CR, PR or SD).

PFS was calculated from the date of registration. The progression time of patients who did not progress at the time of final analysis or who withdrew early without documentation of progression were censored at the time of the last tumor evaluation. OS was calculated from the time of registration until death. The time of death for patients alive at the time of final analysis or lost to follow-up was censored at the last date they were known to be alive. Patients who rolled over to subsequent, companion AGS-003 trials were routinely monitored for survival.

Safety was assessed by monitoring the incidence of adverse events, laboratory assessments, localized injection-site reactions, and changes in size, tenderness, or inflammation of draining lymph nodes throughout the study. Adverse events were assessed by investigators as being possibly, probably, definitely or not related to AGS-003, sunitinib, or the combination, and graded for severity according to the NCI Common Terminology Criteria for Adverse Events (CTCAE) v3.0. Changes from baseline values in clinical laboratory tests and changes in physical examinations, vital signs, and electrocardiograms (ECGs) were determined after study drug dosing. Evaluation for autoimmune reactions was determined by assessing clinical signs and symptoms (i.e.*,* rash, cytopenias, and arthralgias) and by laboratory assessments (i.e., anti-nuclear antibody, rheumatoid factor, anti-double stranded DNA antibody, total hemolytic complement, anti-thyroid antibody, indirect Coombs test).

### Statistical Analysis

The study was designed to enroll approximately 50 patients in order to have a sufficient number of patients who would be eligible to initiate combined AGS-003 plus sunitinib treatment and be evaluable for the assessment of primary and secondary endpoints. The planned analysis for the primary endpoint (complete response rate) required a total 38 evaluable and treated patients for 90% power to detect a CR rate of 20% versus the null rate of 5%, with a type-I error of 5%. No favorable risk patients were enrolled and nearly half of the evaluable patients had 3 or more RCC risk factors (i.e., poor risk) at the time of diagnosis. Study enrollment was stopped early when the sponsor concluded that the initial statistical design and expectations of complete response was inappropriate in the enrolled population of intermediate and poor risk patients. Rather than amend the protocol to a more appropriate primary endpoint such as overall survival, the decision was made to terminate further enrollment, to assess appropriate secondary endpoints, and to plan for a larger randomized study in a similar, intermediate and poor risk mRCC patient population. Prior to early termination of enrollment, twenty-five patients were recruited for study participation.

Response was assessed per RECIST v1.0, however the planned statistical analyses for the primary endpoint were not performed due to the limited enrollment to the study. PFS and OS analyses were performed using the Kaplan-Meier method with two-sided 95% CIs for the medians. SAS version 9.2 and SPLUS version 6.2 were used for statistical analyses. Proc LIFETEST was used for Kaplan-Meier analyses. Relationship between clinical outcome and immune monitoring was analyzed using JMP version 10.0.0.

ECG, vital signs, body weight, and clinical laboratory data (actual values and changes from baseline at each assessment time point) were summarized with descriptive statistics. Laboratory results were evaluated based on laboratory-specified reference ranges and investigator determinations of clinical significance for abnormal results.
